# Effects of Central Injection of Anti-LPS Antibody and Blockade of TLR4 on GnRH/LH Secretion during Immunological Stress in Anestrous Ewes

**DOI:** 10.1155/2014/867170

**Published:** 2014-02-11

**Authors:** Karolina Haziak, Andrzej Przemysław Herman, Dorota Tomaszewska-Zaremba

**Affiliations:** The Kielanowski Institute of Animal Physiology and Nutrition, Polish Academy of Sciences, 05-110 Jabłonna, Poland

## Abstract

The present study was designed to examine the effect of intracerebroventricular (icv) administration of antilipopolysaccharide (LPS) antibody and blockade of Toll-like receptor 4 (TLR4) during immune stress induced by intravenous (iv) LPS injection on the gonadotropin-releasing hormone/luteinizing hormone (GnRH/LH) secretion in anestrous ewes. Injection of anti-LPS antibody and TLR4 blockade significantly (*P* < 0.01) reduced the LPS dependent lowering amount of *GnRH* mRNA in the median eminence (ME). Moreover, blockade of TLR4 caused restoration of *LH-**β*** transcription in the anterior pituitary decreased by the immune stress. However, there was no effect of this treatment on reduced LH release. The results of our study showed that the blockade of TLR4 receptor in the hypothalamus is not sufficient to unblock the release of LH suppressed by the immune/inflammatory challenges. This suggests that during inflammation the LH secretion could be inhibited directly at the pituitary level by peripheral factors such as proinflammatory cytokines and circulating endotoxin as well.

## 1. Introduction

An immune stress inhibits reproductive functions in many animal species and humans [1–4]. Most studies examined the impact of immune stress on reproductive system activity that used bacterial endotoxin lipopolysaccharide (LPS) as a model of infection induced changes. Lipopolysaccharide is a pathogenic membrane component of virtually all Gram-negative bacteria and it is released from the surface of replicating and dying Gram-negative bacteria into the circulation [5]. Bacterial endotoxin is thought to play a major role in the pathophysiology of septic shock [2]. Endotoxin stimulation of animal cells occurs through a signalling cascades with several proteins including CD14 protein, MD-2 protein, and LPS-binding protein (LBP), a necessary component of corresponding LPS receptor called Toll-like receptor 4 (TLR4) [6, 7]. LPS enters the bloodstream associated with LBP. Then, LPS-LBP complex binds to the CD14 protein, which is necessary for the activation of TLR4. CD14, MD-2, and TLR4 as a whole make up the cellular LPS specific receptor [8, 9]. After activation by endotoxin, TLR4 transduces its inflammatory signal through complex intracellular pathways, leading to activation of transcription factors such as nuclear factor kappa-light-chain-enhancer of activated B cells (NF-*κ*B), c-Jun N-terminal kinase (JNK), and protein kinases p38 or inducing cell apoptosis [10, 11].

Administration of LPS inhibits tonic luteinizing hormone (LH) secretion in many species including rats [12], sheep [13], cattle [1], and nonhuman primates [4] as well as delays or completely blocks the preovulatory LH surge [14]. In our earlier studies carried out on anestrous ewes, we showed that suppressive effect of LPS on GnRH/LH secretion occurs primarily at the level of hypothalamus, changing the gonadotropin-releasing hormone (GnRH) synthesis and release as well at the pituitary level by inhibiting release of LH from gonadotropic cells [15, 16].

Data from literature indicate the existence of many possible mechanisms mediating antireproductive action of immune stress. It was shown that the crucial role in the mediation of the inhibitory influence of inflammation on GnRH/LH secretion play the role of proinflammatory cytokines such as IL-1, IL-6, TNF-*α* [17, 18]. The results of our studies showed that IL-1*β* is one of the most important factors in modulating the function of GnRH neurons in anestrous ewes during immune stress [19]. However, cytokine dependent pathway is not only mechanism via an immune challenge that affects the reproduction processes in animals. The studies on ewes in anestrous period indicated the presence of *TLR4* mRNA in the hypothalamic structures such as the preoptic area (POA), the anterior hypothalamic area (AHA), the medial basal hypothalamus (MBH), the median eminence (ME), and in the anterior pituitary gland (AP) in control animals as well as after LPS treatment. The expression of the gene encoding this receptor in ewes treated with LPS was significantly higher than that determined in control animals [20]. It is worth to note that TLR4 expression was found in the central nervous system (CNS) not only in microglia cells, but even in neurons [21]. This suggests that TLR4 and its ligand LPS could be involved in inhibition of the reproductive function at the CNS and directly influence GnRH/LH secretion at the hypothalamic or pituitary level. The question of the possible penetration of endotoxin from blood to the cerebrospinal fluid (CSF) during immune stress and thus direct LPS action at the brain level is still open because the results of *in vivo* studies are not equivocal. The study performed on rats showed that peripherally injected LPS labelled with iodine 125 crossed the blood-brain barrier (BBB). Although the brain uptake of circulating LPS was found to be low, it was measurable [22]. On the other hand, Singh and Jiang [23] suggested that LPS modulates the functioning and permeability of the BBB but does not cross it.

The research hypothesis assumes that LPS given peripherally (intravenously—iv) can directly affect the hypothalamic-pituitary-gonadal axis (HPG) at the CNS level that was verified.

The aim of our experiments was to study the influence of the intracerebroventricular (icv) injection of anti-LPS antibody or blockade of TLR4 receptor during immune stress induced by iv LPS administration on the GnRH/LH secretion and TLR4 gene expression in hypothalamus and pituitary of anestrous ewes.

## 2. Materials and Methods

### 2.1. Animals

The studies were conducted on adult, 3-year-old Polish Longwool ewes in the anestrous season (April-May). All animals were in good condition, and their body condition score was estimated at 3 points (on a five-point scale). The animals were maintained indoors in individual pens and exposed to natural daylight. The ewes were well adapted to the experimental conditions; they always had visual contact with their neighbours, even during the experimental period, to prevent the stress of social isolation. The animals were fed a constant diet of commercial concentrates with hay and water available *ad libitum*. One month before starting of the experiment all groups of ewes were cannulated with stainless steel guide cannulas (1.2 mm o.d.) into the third ventricle under stereotaxic control [24]. The guide cannula was fixed to the skull with stainless steel screws and dental cement. The correct placement of the guide cannula into the third ventricle was established by the efflux of the cerebrospinal fluid from cannula during the surgery. Additionally, the placement of the cannula was checked by inspection of the brain after decapitation. All animals had a venous catheters implanted into jugular vein the day before the experiment.

All experimental procedures were performed in accordance with the Polish Guide for the Care and Use of Animals (1997) and were approved by the Local Ethics Committee of the Warsaw University of Life Sciences.

### 2.2. Experimental Procedures

#### 2.2.1. Inducing Immune Stress in the Experimental Animals

Immune stress was induced in treated animals by intravenous (iv) bolus injection of an appropriate volume of LPS (400 ng/kg body weight) from *E. coli* 055:B5 (Sigma-Aldrich, St. Louis, MO, USA) dissolved in saline (0.9% w/v NaCl) (Baxter, Deerfield, IL, USA) at a concentration of 10 mg/L into the jugular vein. The maximum volume of LPS solution (10 mg/L) administered to any animal was 2.5 mL. The control group received an equivalent volume of saline.

#### 2.2.2. Experimental Schedule

The animals (*n* = 20) were randomly assigned to four experimental groups: (1) “NaCl control group” (*n* = 5), received intracerebroventricular (icv) injection of Ringer-Locke's solution (RLs) into the third ventricle of the brain and 15 min later iv injection of NaCl; (2) “LPS control group” (*n* = 5), received RLs (icv) and 15 min later LPS (iv); (3) “anti-LPS group” (*n* = 5), received monoclonal anti-LPS antibody (Abcam, Cambridge, UK) (icv) in dose of 10 *μ*g/animal and 15 min later LPS (iv); (4) “anti-TLR4 group”, received (icv) antibodies binding TLR4 receptor complex components, anti-LBP (Abcam, Cambridge, UK) in dose of 20 *μ*g/animal, and anti-MD2 (Abcam, Cambridge, UK) in dose of 10 *μ*g/animal and 15 min later LPS (iv). All antibodies were dissolved in sterile RLs.

Jugular blood samples from each ewe were taken for LH and cortisol measurement at 15 min intervals, beginning 2 h before endotoxin or saline injection (iv) preceded by the injection of appropriate substances (icv), and continuing 4 h after LPS or saline treatment.

*Effect of Immune Stress on the Gene Expression in Hypothalamic Structures and in the AP*. After 2 weeks of convalescence, the same animals (*n* = 20) were used. Hypothalamic structures (the preoptic area—POA, the anterior hypothalamic area—AHA, the medial basal hypothalamus—MBH, and the median eminence—ME) and the anterior pituitary glands (AP) were collected 2 h after iv injection of LPS or saline preceded by the icv injection of corresponding substances as described above. The animals were slaughtered by decapitation, the brains were rapidly removed from the skulls, and then chosen hypothalamic structures and APs were dissected. All tissues were frozen immediately after collection in liquid nitrogen and were stored in −80°C until assay.

### 2.3. Assays

#### 2.3.1. Radioimmunoassay for LH

The concentration of LH in plasma was assayed by the radioimmunoassay (RIA) double-antibody method using anti-ovine-LH and anti-rabbit-*γ*-globulin antisera and ovine standard (NIH-LH-SO18) as described by Stupnicki and Madej [25]. The sensitivity was 0.3 ng/mL; intra-assay and interassay coefficients of variation were 8.3% and 12.5%, respectively.

#### 2.3.2. Radioimmunoassay for Cortisol

The cortisol concentrations were determined by the RIA method according to Kokot and Stupnicki [26], using rabbit anticortisol antisera (R/75) and HPLC grade cortisol standard (Sigma). The assay sensitivity was 0.95 ng/mL and the intra-assay and interassay coefficients of variation were 10% and 12%, respectively.

#### 2.3.3. Relative Gene Expression Assays

Total RNA from hypothalamic and pituitary tissues was isolated using NucleoSpin RNA II Kit (MACHEREY-NAGEL Gmbh & Co, Düren, Germany) according to manufacturer's protocol. The purity and concentration of isolated RNA were quantified spectrophotometrically by measuring the optical density at 260 and 280 nm in a NanoDrop 1000 instrument (Thermo Fisher Scientific Inc., Waltham, USA). The RNA integrity was verified by electrophoresis using 1% agarose gel stained with ethidium bromide. DyNAmo SYBR Green 2-Step qRT-PCR Kit (Finnzymes, Espoo, Finland) was used to prepare cDNA synthesis. As a starting material for this PCR synthesis 800 ng of total RNA was used.

Real-time RT-PCR was carried out using SYBR Green 2-Step qRT-PCR Kit (Finnzymes, Espoo, Finland) components and HPLC-grade oligonucleotide primers synthesized by Genomed (Poland). Specific primers for determining the expression of housekeeping genes and the genes of interest (Table 1) were designed using Primer 3 software. One tube contained 10 *μ*L PCR Master Mix (2x), 7 *μ*L RNase-free water, 2 *μ*L primers (1 *μ*L each, working concentration was 0.5 *μ*M), and 1 *μ*L cDNA template. The tubes were run on the Rotor-Gene 6000 (Qiagen, Duesseldorf, Germany). The following protocol was used: 95°C in 15 min for activating Hot Start DNA polymerase and finally the PCR including 30 cycles at 94°C in 5 sec for denaturation, 56°C in 20 sec for annealing, and 72°C in 15 sec for extension. After the cycles, a final melting curve analysis under continuous fluorescence measurements was performed to confirm the specificity of the amplification.

### 2.4. Data Analysis

#### 2.4.1. Plasma Hormones Concentration Data Analysis

All data are presented as hormone concentration expressed as mean ± SEM. The results of LPS treatments on the concentrations of plasma LH and cortisol were examined by two-way analysis of variance—ANOVA (STATISTICA; Stat-Soft, Inc., Tulsa, OK, USA) to identify treatment effects and significant interactions between the control and experimental groups. All experiments consisted of a baseline period when no treatment was given (−2 to 0 h before) and period when treatments were applied (+1 to +4 h after LPS or saline injection). Data was integrated over time. ANOVA for hormone parameters excluded data during the first hour after LPS or saline treatment to allow time for treatments to take effect. When a significant treatment by time interaction was observed, *post hoc* analysis was conducted to identify treatment effects. The Mann-Whitney *U* test was used to compare pre- versus posttreatment values.

#### 2.4.2. PCR Data Analysis

Relative gene expression was calculated using the comparative quantification option of Rotor Gene 6000 software 1.7 (Qiagen, Duesseldorf, Germany). The second differential maximum method [27] was used in this analysis to calculate reaction efficiencies and a set percentage of the maximum fluorescence value to calculate the beginning of the exponential phase. To compensate a variation in cDNA concentrations and the PCR efficiency between tubes, an endogenous control gene was assayed in each sample and used for normalization. Initially, three housekeeping genes glyceraldehyde-3-phosphate dehydrogenase (GAPDH), *β*-actin (ACTB), and cyclophilin C (PPIC) were tested. The BestKeeper was used to determine the most stable housekeeping gene, for normalizing genes of interest expression. The BestKeeper was based on the pair-wise correlation analysis of all pairs of candidate genes [28] and calculates variations of all reference genes (SD (±Ct)). PPIC was chosen as the best endogenous control gene. It had the lowest SD (±Ct) value and a good correlation coefficient with the remaining analysed housekeeping genes.

The results are presented as relative gene expression of the target gene versus housekeeping gene, relative expression value and mean ± SEM. The average relative quantity of gene expression in control groups was set to 1.0. The significance of differences between the experimental groups was assessed by the Mann-Whitney *U* test.

## 3. Results

### 3.1. Effect of Central Injection of Anti-LPS Antibody and Blockade of TLR4 on Cortisol Release during LPS-Induced Inflammation

**Figure 1:**
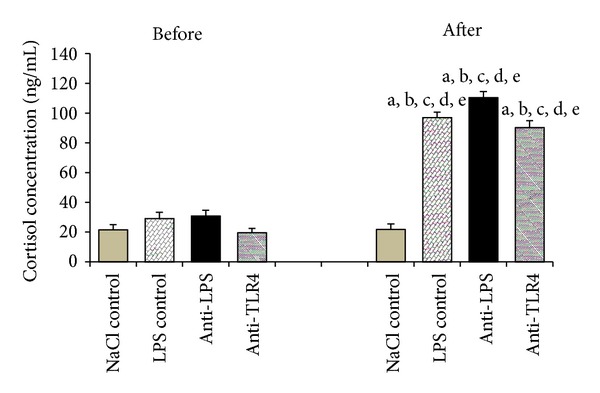
The effect of central injection of LPS antibody and blockade of TLR4 on cortisol release before and after LPS or saline treatment. Data are presented as a mean value ± SEM; letters indicate values that differ significantly according to the Mann-Whitney *U* test from “NaCl control before” (^a^ *P* < 0.01); “NaCl control after” (^b^ *P* < 0.01); “LPS control before” (^c^ *P* < 0.01); “anti-LPS before” (^d^ *P* < 0.01); “anti-TLR4 before” (^e^ *P* < 0.01), respectively.

LPS administration significantly (*P* < 0.01) increased plasma cortisol level in all LPS-treated groups (LPS control, anti-LPS, anti-TLR4) compared with NaCl control group (Figure 1).

### 3.2. Effect of Central Injection of Anti-LPS Antibody and Blockade of TLR4 on LH Secretion during LPS-Induced Inflammation

**Figure 2:**
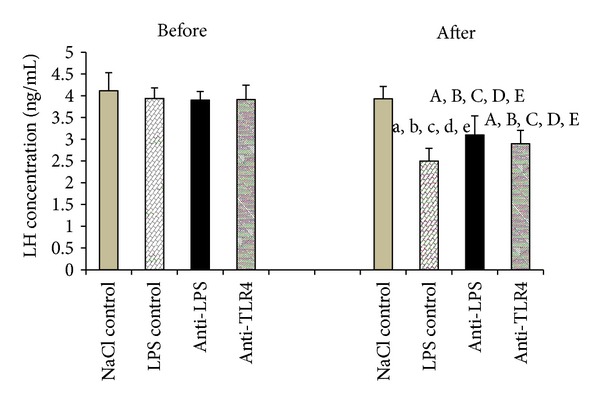
The effect of central injection of LPS antibody and blockade of TLR4 on LH release before and after LPS or saline treatment. Data are presented as a mean value ± SEM. Letters indicate values that differ significantly according to the Mann-Whitney *U*-test from “NaCl control before” (^a^ *P* < 0.01; ^A^ *P* < 0.05); “NaCl control after” (^b^ *P* < 0.01; ^B^ *P* < 0.05); “LPS control before” (^c^ *P* < 0.01; ^C^ *P* < 0.05); “anti-LPS before” (^d^ *P* < 0.01; ^D^ *P* < 0.05); “anti-TLR4 before” (^e^ *P* < 0.01; ^E^ *P* < 0.05), respectively.

Intravenous injection of LPS significantly reduced plasma LH release in all LPS-treated groups (LPS control: *P* < 0.01; anti-LPS: *P* < 0.05; anti-TLR4: *P* < 0.05) compared with the saline control. The central administration of anti-LPS as well as icv injection of anti-LBP and anti-MD2 did not influence on lowered LH secretion (Figure 2).

**Figure 3:**
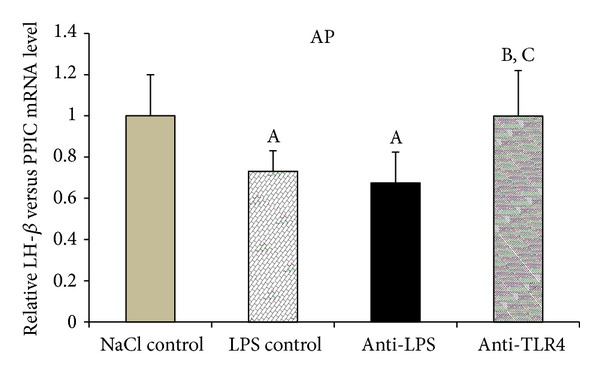
The effect of central injection of LPS antibody and blockade of TLR4 on the relative *LH-*β** mRNA level in the anterior pituitary gland during LPS-induced inflammation. Data are presented as a mean value ± SEM. Letters indicate values that differ significantly according to the Mann-Whitney *U* test from “NaCl control” (^A^ *P* < 0.05); “LPS control” (^B^ *P* < 0.05); “anti-LPS” (^C^ *P* < 0.05), respectively.

Peripheral administration of endotoxin significantly (*P* < 0.05) decreased the gene expression of *LH-*β** in the AP in LPS control and anti-LPS groups compared with NaCl control group. On the other hand, injection of antibodies binding TLR4 receptor complex components anti-LBP and anti-MD2 (anti-TLR4 group) released* LH-*β**gene expression from the suppressive effect of LPS administration (Figure 3).

### 3.3. Effect of Central Injection of Anti-LPS Antibody and Blockade of TLR4 on* GnRH *and *GnRH-R *Genes Expression during LPS-Induced Inflammation

**Figure 4:**
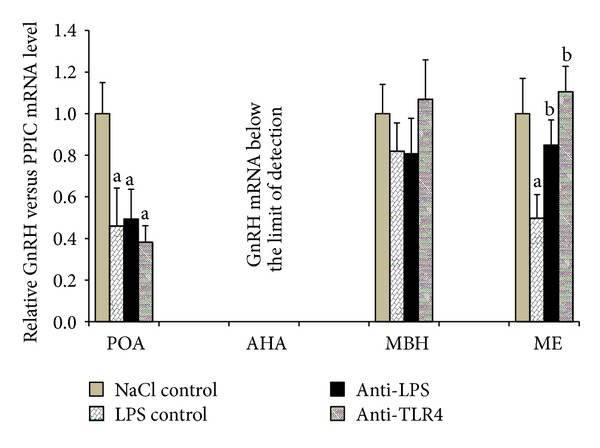
The effect of central injection of LPS antibody and blockade of TLR4 on the relative *GnRH* mRNA level in the hypothalamic structures (POA, AHA, MBH, and ME) during LPS-induced inflammation. Each point represents mean ± SEM. Letters indicate values that differ significantly according to the Mann-Whitney *U* test from “NaCl control” (^a^ *P* < 0.01); “LPS control” (^b^ *P* < 0.01), respectively.

Injection of LPS significantly (*P* < 0.01) decreased *GnRH *gene expression in LPS control group from hypothalamic structures such as the POA (by 54%) and the ME (by 50%) compared with NaCl control group. In the ME, the central administration of anti-LPS antibody (anti-LPS group) and antibodies binding TLR4 receptor complex components anti-LBP and anti-MD2 (anti-TLR4 group) significantly (*P* < 0.01) reduced LPS dependent suppression of *GnRH *gene expression compared with LPS control group. No effects of iv and icv treatments on *GnRH* expression were found in the MBH. The amount of *GnRH* mRNA determined in the AHA was too low and did not enable the quantitative analysis in this hypothalamic structure (Figure 4).

**Figure 5:**
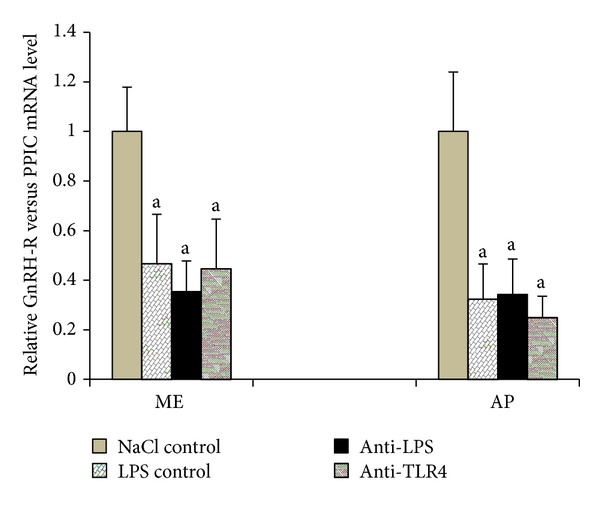
The effect of central injection of LPS antibody and blockade of TLR4 on the relative *GnRH-R* mRNA level in the ME and the AP during LPS-induced inflammation. Each point represents mean ± SEM; “^a^” indicate values that differ significantly according to the Mann-Whitney *U* test from “NaCl control” (*P* < 0.01).

Gene expression of receptor for *GnRH-R* significantly (*P* < 0.01) decreased in LPS control groups in the ME and in the AP compared with NaCl control group. Central administration of anti-LPS or antibodies binding TLR4 receptor complex components anti-LBP and anti-MD2 did not affect *GnRH-R *gene expression compared to LPS control group (Figure 5).

### 3.4. Effect of Central Injection of Anti-LPS Antibody and Blockade of TLR4 on* TLR4 *Gene Expression during LPS-Induced Inflammation

**Figure 6:**
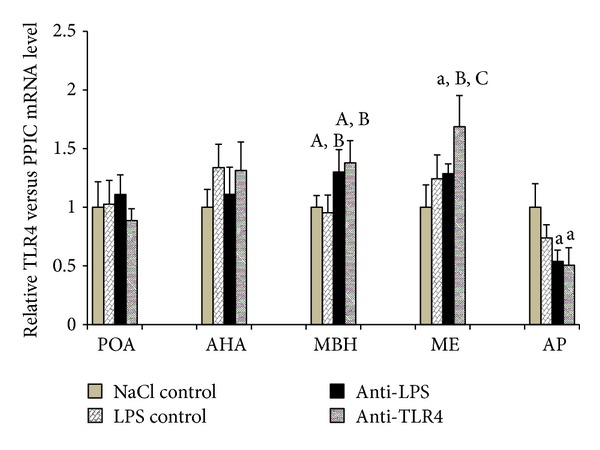
The effect of central injection of LPS antibody and blockade of TLR4 on the relative *TLR4* mRNA level in the hypothalamic structures (POA, AHA, MBH, and ME) and in the AP during LPS-induced inflammation. Each point represents mean ± SEM. Letters indicate values that differ significantly according to the Mann-Whitney *U* test from “NaCl control” (^a^ *P* < 0.01; ^A^ *P* < 0.05); “LPS control” (^B^ *P* < 0.05); “anti-LPS” (^C^ *P* < 0.05), respectively.

*TLR4 *gene expression was detected in four analysed hypothalamic structures and in the AP. Concomitant administration of LPS (iv) and anti-LPS (icv) increased (*P* < 0.05) mRNA *TLR4* level in the MBH but decreased (*P* < 0.01) it in the AP compared with NaCl control group. It has been shown that the administration of LPS (iv) together with antibodies binding TLR4 receptor complex components anti-LBP and anti-MD2 (icv) increased *TLR4 *gene expression in the ME compared with the other groups (NaCl control: *P* < 0.01; LPS control: *P* < 0.05; anti-LPS: *P* < 0.05). An increase (*P* < 0.05; *P* < 0.05) of *TLR4 *gene expression in anti-TLR4 group was also demonstrated in the MBH compared with NaCl control and LPS control groups, respectively, whereas a decrease (*P* < 0.01) in the AP compared with NaCl control group. In the POA and the AHA observed differences between analysed groups were not significant (Figure 6).

## 4. **Discussion**

Our study shows the inhibitory effect of LPS-induced immune stress on LH secretion in sheep which is consistent with previous *in vivo* studies conducted on anestrous ewes [15, 16]. Other researchers also showed that LPS affects LH secretion and even disturbs the preovulatory LH surge in ewes [29, 30]. In castrated rams, LPS significantly reduced plasma LH level and the number of LH pulses [2]. Likewise, Refojo et al. [12] demonstrated that endotoxin lowered LH concentrations by inhibiting several pulsatility parameters such as frequency, amplitude, and maximum values in male rats.

The most important regulator of LH secretion is GnRH, which affects reproduction processes at level of the CNS by stimulation of the gonadotrophs in the AP to secrete LH. Functional regulation of LH secretion is mediated by the pulsatile secretion of GnRH into the hypophyseal portal vasculature [31]. It has been demonstrated that pulsatile pattern of LH secretion is a direct reflection of GnRH secretion from hypothalamus in ovariectomized ewes [32]. Changes in LH secretion observed after peripheral injection of LPS suggest that immune stress acts on the reproductive functions at the level of the hypothalamus through alterations of GnRH secretion. Fergani et al. [33] showed that peripheral endotoxin administration caused disorders in the GnRH/LH surge. It could be caused by less GnRH release or that pituitary responsiveness to GnRH may have been comprised. Previous studies on the ovine model clearly showed that administration of bacterial endotoxin induced an immune/inflammatory stress and reduced pulsatile GnRH secretion [2, 13].

In presented study, bacterial endotoxin lowered *GnRH* mRNA level in the POA, where more than half of all GnRH perikarya are located [34]. It was determined that *GnRH *mRNA level in the POA was the highest among other analysed hypothalamic structures, which confirms earlier reports that neurons located in the POA synthesized most of *GnRH* transcripts in anestrous phase ewes [15]. This fact further supports the assumption that activity of GnRHergic neurons in the hypothalamus is modulated by immune stress. However, it has not been proven that immune challenge affects the GnRH synthesis at the transcriptional or posttranscriptional levels. Observed changes in *GnRH *mRNA content in the hypothalamic area may not result from decreased *GnRH* gene transcription which is fairly stable [35] but from lowered accumulation or increased degradation cytoplasmic *GnRH* mRNA. This data suggests that the suppressive effect of immune stress on GnRH release to the hypophyseal portal blood previously described in sheep [36] could result from reduced *GnRH* gene expression in the POA. It is worth to mention that in the present study there were no significant changes in *GnRH *gene expression in the MBH and the amount of *GnRH* mRNA in the AHA was below the limit of detection, which is consistent with previously obtained results [15] and suggests that in anestrous ewes GnRH neurons located in these hypothalamic structures do not play pivotal role in communication between neuroendocrine and immune systems. It was also found that LPS significantly decreased *GnRH* gene expression in the ME, where GnRH neurons terminals are located. This phenomenon has been described in detail in our previous study [15] and it has been suggested that the selective transport of *GnRH* transcript to the distal part of neurons occurred in the GnRHergic neurons. Decreased content of GnRH in the ME after LPS treatment could result from decreased transport of *GnRH* mRNA to the nerves terminals as well as increased degradation of *GnRH* transcript in this structure. It was previously found that a gradual reduction of the poly(A) tail of mRNA occurs during its translocation from the perykaryon to the nerve terminal [37–39]. Therefore, it may be assumed that mRNA stored in nerves terminals is more sensitive to all factors affecting the stability of these transcripts than mRNA that occurs in the region of the neuronal body.

In the present study, decreased *GnRH-R* gene expression after LPS administration was determined in the AP and ME. It supports the previous studies carried out on ovariectomized ewes that immune stress lowered *GnRH-R* gene expression in the pituitary gland [40]. Similar results were also obtained in rats where the administration of LPS affected the GnRH-R expression both in hypothalamus and pituitary [41]. The decrease in the level of *GnRH-R* mRNA in the AP may be due to lower gonadotrophs stimulation by GnRH, which is the main factor controlling the amount of its receptors [42]. In turn, the reduction of *GnRH-R* gene expression may lead to a decrease GnRH-R expression in gonadotrophs and lower sensitivity of these cells on GnRH stimulation. This may lead to lower LH-*β* synthesis in the AP.

In the presented study, the injection of endotoxin significantly increased the plasma level of cortisol. It fully supports the previous studies reported about stimulatory effect of immune stress on cortisol release in various animal species including sheep [2, 16, 20, 43]. The elevation of the cortisol release suggests the activation of the hypothalamic-pituitary-adrenal (HPA) axis, which may result in inhibition of the HPG axis [2, 43]. Immune challenge stimulates the synthesis of HPA axis components, such as arginine vasopressin, corticotropin-releasing hormone (CRH), adrenocorticotrophic hormone, and corticosterone/cortisone from adrenal cortex [44, 45]. All these factors have an inhibitory effect on the HPG axis [46, 47]. However, the role of cortisol and other HPA axis components in the suppression of the GnRH/LH secretion during immune stress seems to be ambiguous. Rivest and Rivier [48] demonstrated in rats that reproductive system inhibited by LPS injection has not been released from its suppressive action by CRH antibodies administration, although this administration prevented the increase in the HPA axis activity. In study carried out on sheep, Debus et al. [43] demonstrated that the cortisol secretion blockage did not lower the suppression of GnRH/LH release caused by LPS treatment.

One of the mechanisms through endotoxin that may modulate the neuroendocrine system is induction of pro-inflammatory cytokines [23, 49]. However, the main source of centrally acting cytokines seems to be their local synthesis in the brain parenchyma [50]. These cytokines can be also secreted by the BBB cells activated by endotoxin [23, 51, 52] as well as the choroid plexus cells [53]. It is worth mentioning that some amounts of the central cytokines could have a peripheral origin and cross the BBB due to the existence of saturated transport mechanism [54]. Another possible pathway of endotoxin penetration to the brain is through the organum vasculosum laminae terminalis (OVLT), which is one of the sensory circumventricular organs, forming the anterior wall of the third ventricle [55]. This structure is devoid of the BBB, so OVLT could be a potential location for LPS bypassing into the brain parenchyma. A direct response of OVLT cells to exposure to endotoxin or cytokines was demonstrated by Ott et al. [56]. Their study showed that the OVLT cells secrete proinflammatory cytokines (e.g., TNF-*α*, IL-1*β*, and IL-6). It has been previously reported that LPS acting indirectly via stimulation of central cytokines synthesis affects GnRH secretion in the hypothalamus and can disturb LH secretion from the AP [13, 18, 19].

On the other hand, one of the mechanisms by which peripherally administered endotoxin affects central response is the activation of the afferent vagal nerves by prostaglandins (PGs), other important regulatory factors of GnRH/LH levels suppression during immune stress [57]. Rettori et al. [58] showed that inhibition of prostaglandin E_2_ (PGE_2_) suppressed the release of GnRH/LH. This inhibition could be caused via PG-dependent pathways. Peripheral administration of LPS induces synthesis of endogenous cytokines (e.g., IL-1*β*) and activates the projection area of the vagal nerves in the brain [59]. Presence of receptors for IL-1 was demonstrated in study of Ek et al. [60] which suggested that IL-1*β* stimulates vagal sensory activity. This activation of afferent nerve fibers by peripherally released cytokines could be a fast pathway of immune signals transfer from the periphery to the brain. However, in response to circulating cytokines, a slow humoral pathway of transmission is activated [61]. Immune challenge could act as well in this PG-dependent manner represented by PGs synthesis by cyclooxygenase-2 (COX-2) around blood vessels [62]. These observations suggest that PGs play a role in mediating between the immune and neuroendocrine systems [57, 63].

In our study, *TLR4* gene expression was determined in the hypothalamic structures such as the POA, AHA, MBH, and ME and in the AP. However, no effect of LPS administration on *TLR4 *transcription in all these structures was observed. These results are partially contrary to our previous study performed on anestrous ewes [20] where significant increase of the *TLR4* gene expression was determined. The existence of TLR4 receptor in the hypothalamus may suggest the possible direct action of LPS in the CNS. Although experiments carried out on cats [64] seem to exclude the penetration of endotoxin from the blood to the brain, the results of experiments conducted on rats are inconclusive. Singh and Jiang [23] suggest that LPS modulates the permeability of the BBB but does not exceed it. However, *in vivo* research performed on mice [22] and rats [65] have shown that iodine-radiolabelled LPS penetrated the BBB in measured quantities. The study performed on rats showed that central administration of endotoxin suppressed the secretion of LH in rats [66]. This proves the potential of centrally acting LPS to suppress the HPG activity at the hypothalamic level. In present study, it was determined that the blockade of TLR4 receptor in the hypothalamus as well as administration of anti-LPS antibody into the region of hypothalamus reverses decreasing effect of LPS treatment on *GnRH* mRNA level in the ME. However, no effects of these treatments were observed in the structures where GnRH neurons perikarya are located. The fact that the blockade of TLR4 receptor as well as administration of anti-LPS antibody into the third ventricle restored *GnRH* mRNA content only in the ME suggests that these treatments prevented the inflammation, dependent decreasing of the *GnRH* mRNA stability rather than decreasing *GnRH* gene transcription. The inhibition of TLR4 receptor as well as decreasing the number of its interacting ligand could result in decreased proinflammatory cytokines synthesis in the hypothalamus. It was previously suggested that acting in the region of hypothalamus proinflammatory IL-1*β* could be responsible for decreasing the stability of *GnRH* mRNA and reduction of its translation [19].

In our study, restoration of *LH-*β** mRNA content to the control level was observed only in the anti-TLR4 group. However, this change in the *LH-*β** gene expression was not accompanied by the elevation of the circulating LH concentration. The lack of parallelism between the increased LH transcription and the peripheral level of LH in the anti-TLR4 group suggests that LH release was still inhibited by the peripheral immune/inflammatory challenges affecting the HPG axis at the pituitary level. This suppression may result from the action of proinflammatory cytokines whose receptors are widespread in the pituitary gland [67]. The results of our *ex vivo* study showed that IL-1*β* is a potent downregulator of LH secretion directly from the pituitary and suggested that this direct action of interleukin could have a profound effect on the suppression of LH release occurring during an inflammatory state [68]. The *in vitro* study performed on the mouse AtT-20 pituitary tumor cells showed that direct LPS treatment increases the number of IL-1R1 in a dose-dependent manner [69]. The studies carried out on mice [70] and sheep [71] also reported the stimulating effect of LPS on IL-1R1 mRNA. Another inflammatory cytokine involved in direct modulation of the secretory activity of the pituitary is IL-6. The *in vitro* study showed that IL-6 significantly suppressed GnRH-stimulated LH release from male rats dispersed pituitaries throughout the dose range but did not influence basal LH release [72]. It is worth mentioning that stress caused by LPS injection may increase the number of cytokines receptors expressed in the AP [73]. The factor suppressing the LH secretion at the level of pituitary could be also LPS itself. Our previous *ex vivo* study showed that LPS directly decreases LH secretion from the ovine AP explants [74]. It was suggested that the secretion of LH from the pituitary could be affected directly by LPS and/or could result from autocrine action of proinflammatory cytokines secrete by the folliculostellate cells.

## 5. **Conclusions**

The study suggests that the blockade of TLR4 receptor in the hypothalamus during LPS-induced immune stress restores the *LH-*β** transcription in the pituitary gland. However, this treatment is not sufficient to unblock the release of LH suppressed by the peripheral immune/inflammatory challenges.

## Figures and Tables

**Table 1:** Specific primers used in real-time PCR for determining the expression of housekeeping genes and genes of interests.

Gene bank acc. number	Gene	Amplicon size [bp]	Forward/reverse	Sequence 5′→ 3′
NM_001034034	*GAPDH* glyceraldehyde-3-phosphate dehydrogenase	134	Forward	AGAAGGCTGGGGCTCACT
			Reverse	GGCATTGCTGACAATCTTGA

U39357	*ACTB* beta actin	168	Forward	CTTCCTTCCTGGGCATGG
			Reverse	GGGCAGTGATCTCTTTCTGC

NM_001076910	*PPIC* cyclophilin C	131	Forward	ACGGCCAAGGTCTTCTTTG
			Reverse	TATCCTTTCTCTCCCGTTGC

U02517	*GnRH * gonadotropin-releasing hormone	123	Forward	GCCCTGGAGGAAAGAGAAAT
			Reverse	GAGGAGAATGGGACTGGTGA

X52488	*LH-*β** luteinizing hormone beta-subunit	184	Forward	AGATGCTCCAGGGACTGCT
			Reverse	TGCTTCATGCTGAGGCAGTA

NM_001009397	*GnRH-R * gonadotropin-releasing hormone receptor	150	Forward	TCTTTGCTGGACCACAGTTAT
			Reverse	GGCAGCTGAAGGTGAAAAAG

AY957615	*TLR4* Toll-like receptor 4	117	Forward	GGTTCCCAGAACTGCAAGTG
			Reverse	GGATAGGGTTTCCCGTCAGT
